# Intention for Car Use Reduction: Applying a Stage-Based Model

**DOI:** 10.3390/ijerph15020216

**Published:** 2018-01-26

**Authors:** Lars E. Olsson, Jana Huck, Margareta Friman

**Affiliations:** CTF Service Research Center and Department of Social and Psychological Studies, Karlstad University, SE-65188 Karlstad, Sweden; jana.huck@kau.se (J.H.); margareta.friman@kau.se (M.F.)

**Keywords:** attitudes, intentions, norms, perceived behavioral control, stage-based models, sustainable travel, work commute

## Abstract

This study investigates which variables drive intention to reduce car use by modelling a stage of change construct with mechanisms in the Theory of Planned Behavior (TPB) and Norm Activation Model (NAM). Web questionnaires (*n* = 794) were collected via 11 workplaces. The socio-demographics, work commute, stage of change, attitudes to sustainable travel modes, social norms, perceived behavioral control, and personal norm were assessed. An initial descriptive analysis revealed that 19% of the employees saw no reason to reduce their car use; 35% would like to reduce their car use but felt it was impossible; 12% were thinking about reducing their car use but were unsure of how or when to do this; 12% had an aim to reduce current car use, and knew which journeys to replace and which modes to use; and 23% try to use modes other than a car for most journeys, and will maintain or reduce their already low car use in the coming months. A series of Ordered Logit Models showed that socio-demographic variables did not explain the stage of change. Instead, personal norms, instrumental and affective attitudes, and perceived behavioral control toward sustainable travel modes were all significant and explained 43% of the variance in stage of change. Furthermore, it was found that the significant relationships were not linear in nature. The analysis also showed an indirect effect of social norms on the stage of change through personal norms. Implications are discussed regarding the design of interventions aimed at influencing a sustainable work commute.

## 1. Introduction

Between 2010 and 2013, there was a 16% increase in the number of registered vehicles in the world according to the WHO [1], with the number of cars on the road globally being predicted to nearly double by 2040 [2]. The documented negative impacts of car use, such as noise, congestion, pollution, and a sedentary lifestyle, provide compelling reasons to adopt and maintain sustainable travel behavior [3]. However, more knowledge is required regarding the mechanisms underlying the intention to change from a car commute to a sustainable work commute (e.g., walking, cycling, using public transport or carpooling) [4]. A better understanding of the process of change would help to clarify the posited theoretical relations and, more importantly, facilitate the development of interventions aimed at addressing and potentially reducing the car commute. This paper applies constructs from stage-based models of behavior change (Transtheoretical Model of Change, TTM [5,6]; Stage Model of Self-Regulated Behavioral Change, SSBC [7,8]) as an analysis framework with the aim of increasing our understanding of the mechanisms underlying the intention to reduce work commutes by car.

From previous research, it is well known how different psychological mechanisms relate to the intention (or motivation) to perform a particular behavior [9]. These psychological mechanisms are defined in the Theory of Planned Behavior [10,11,12] and often applied to travel research. This theory explains how attitudes, social norms, and perceived behavioral control are essential mechanisms for the intention to perform a behavior. Attitudes are psychological tendencies that stem from the evaluation of a specific entity [13]. Perceived behavioral control includes self-efficacy and refers to beliefs about one’s own ability to perform a behavior [14]. Social norms inform people about the behavioral standards that are adequate within their reference group [15]. In addition, the Value-Belief-Norm Theory [16] and the Norm Activation Model [17,18] add personal norms as an important mechanism [19,20,21]. Personal norms are defined either as moral obligations or a sense of duty regarding how to act [17]. Previous research indicates that, taken together, attitudes, perceived behavioral control, and norms generally account for 50 to 70% of the variance in intentions [9,22]. Based on the Theory of Planned Behavior (TPB), Norm Activation Model (NAM), and stage-based intention models, this study contributes to the literature on sustainable travel behavior change. In the following subsection, each of the mechanisms is discussed in relation to the intention to travel. This is followed by a presentation of TTM and SSBC, which has rarely been used in travel behavior research.

### Theoretical Concepts

An attitude can be defined as “… a psychological tendency that is expressed by evaluating a particular entity with some degree of favor or disfavor” [13]. Gärling et al. [23] explain that the particular entity or “attitude object” may be everything a person discriminates against perceptually or keeps in mind. Thus, an attitude toward travel is often made up of perceptions of travel features (e.g., speed, reliability, comfort, and price) in conjunction with individual, social, and societal factors [23]. An attitude can thus have an affective or instrumental character. Attitudes toward travel have been shown to explain up to 54% of the intention (defined as a subjective probability or likelihood) to use a certain travel mode [21].

Perceived behavioral control can affect behavior directly and/or indirectly depending on the degree of matching between beliefs and actual control [22]. A higher degree of matching increases the probability of carrying out the behavior [9], whereas a lower degree of control increases the likelihood of not performing the behavior [24]. The significance of behavioral control seems to vary with transport mode [25,26] and habits [27]. The intentions of people with strong travel habits are less influenced by behavioral control [27,28].

Social norms influence behavior based on the social environment [15]. There is a difference between injunctive and descriptive norms [29,30,31], whereby social injunctive norms refer to whether a behavior is approved of or disapproved of with reference to others. Thus, the injunctive norm implies whether a behavior ought to be performed or not. Descriptive norms, on the other hand, relate to whether or not others (the social reference group) perform the normative behavior. Both injunctive and descriptive norms are important for supporting environmentally friendly behavior [17,30,31]. The more a person identifies with a reference group, the greater the effect of social norms on travel [24] since social norms bring social pressures to behave in a certain way [25]. However, empirical evidence shows that social norms have a marginal direct effect on the intention to change travel behavior [22,25,26]; rather, there is an indirect effect mediated by personal norms [21,32].

Personal norms are an internalization of social norms whereby feelings of guilt or pride are activated and regulate the individual [33]. Personal norms are related to awareness of the consequences associated with a specific behavior. Individuals with an awareness of the negative effects of car use have been found to have a stronger intention to reduce this behavior [25,26,34].

Behavior change is argued to be a progression through a sequence of qualitatively different stages or degrees of motivation [5], also implying that the impact of psychological mechanisms can vary with the degree of intention to change. The Transtheoretical Model of Change [6,35] proposes a five-stage process consisting of precontemplation, contemplation, preparation, action, and maintenance. Precontemplation includes an unawareness of the negative consequences of the current behavior and no intention to change within the coming six months. Contemplation encompasses an awareness of a harmful behavior and the consideration of change. Preparation incorporates concrete plans and initial attempts to try out a new behavior; for example, biking one day a week or for part of the overall distance. Action includes the full initiation of a new behavior; for example, biking every day of the week, or for the entire distance. Finally, maintenance is characterized by the upholding of new habits. Relapsing to previous stages may occur at any time during the process, indicating, for instance, that a new habit may be interrupted, with the process regressing to the action or preparation stages. Depending upon which stage an individual is at, at a specific point in time, different interventions and actions may differ in their effectiveness [36,37].

Similar stage-based ideas have been suggested for pro-environmental behavior, and specifically for travel behavior research where Bamberg introduced the Stage Model of Self-Regulated Behavioral Change, SSBC [7,8]. In this model, four stages are suggested and labelled predecisional, preactional, actional, and postactional. In the predecisional stage the problem behavior is performed on a regular habitual basis, the negative consequences associated with this behavior are not fully known, and they see no reasons for behavioral change. In the preactional stage the general goal of changing current behavior is present, but individuals still face the task of selecting the most personally suitable behavioral strategy. In the actional stage a strong goal intention is present, and a decision on which new behavioral strategy to test (instead of the old one) has been made. In the final, postactional, stage the selected new behavior has been performed for some time, and the person reflects on the experiences related to the new behavior, and compares it critically with the old behavior.

As described above, the stages in TTM and SSBC include very similar features, yet they differ in the number of stages, and in which stage a specific feature should be placed. There is an ongoing debate around the validity of stage-based models, where some critique has been related to the classifications and the number of stages in such models, and some to the fact that stages might differ between contexts [38,39]. It is therefore essential to empirically test stage-based models in a variety of contexts. Changes towards sustainable travel is one such context of great relevance that the present study attempts to address.

In the travel behavior domain, Forward [40] was able to confirm a stage-based model for the intention to bike. She showed how the combination of psychological mechanisms differed from stage to stage. For instance, the attitude toward biking and perceived behavioral control had a stronger impact during the earlier stages. The descriptive social norm was found to be moderate during precontemplation but stronger during the contemplation stage, whereas the subjective social norm did not differ between stages. Gatersleben and Appleton [41] also found that with increasing intention to bike, attitudes became more positive. With respect to car use reduction, stage-based diagnostic measures aimed directly at car use have been developed and tested [7]. Bamberg assessed these diagnostics in his time-dependent framework of SSBC, and confirmed distinctive stages by latent class cluster analysis. Furthermore, the results indicated that personal norms may be an additional psychological mechanism at play [7,8]. Although not applying a stage-based construct of intention, Liu et al. [42] found recently, in a Chinese sample, that the psychological constructs of TPB and NAM do affect the intention to reduce travel by car significantly. Specifically, they found that perceived behavioral control and attitudes had a stronger relationship with intention, and that personal norms had a weaker, although significant, relationship. Social norms were also found to be of importance, but were additionally mediated by personal norms.

The present study will add to previous literature by investigating how psychological mechanisms drive the intention to change travel mode in a stage-based framework. More specifically, we integrate stage of change constructs with the psychological mechanisms proposed by the Theory of Planned Behavior and the Norm Activation Model (attitudes, perceived behavioral control, social norms, and personal norm) to explain car users’ stage-based intentions to change their travel behavior. We also test the potential indirect effects of social norms on the stage of intention to change.

## 2. Materials and Methods 

### 2.1. Participants and Procedure

A web-based questionnaire was distributed within four private companies and seven public institutions throughout the County of Värmland, Sweden. Three of the private companies were engineering and business consultancies, while one is a co-working platform with small businesses and start-ups in various industries. Among the public institutions were one regional public transport organization, one hospital and five municipalities. The municipalities work with a variety of tasks; for example, organization, operation and development of infrastructure and services for residents. A link was sent to a random sample of employees using their corporate email addresses. The emails were sent by our contacts within each organization. The respondents had one month to answer. During this time, two reminders were sent out to those that had not responded to the survey. The respondents received no rewards; however, after completion, they were able to sign up for the chance of a 1-month free trial on regional public transport supported by the regional public transport provider. The responses were collected between October 2016 and February 2017. The questionnaires were adapted to the respective organization using a catalogue of questions. The questions about our target measures were, however, the same in all the questionnaires. We obtained 1132 valid cases. The response rate per workplace ranged from 37 to 81%. The average response rate was 67%.

### 2.2. Questionnaire

The web-based questionnaire consisted of five sections and took approximately 10 min to complete. The questionnaire was completed using a web-based survey tool and followed a simple, comprehensive structure. The questionnaire included the following modules: (1) questions about socio-demographic variables including sex, age, income, household type, access to car and use, access to bike and public transport, distance to worksite; (2) questions about travel habits and choice of mode; (3) questions about psychological mechanisms and intentions related to commuting by car, including the stage of change, attitudes (instrumental, affective), personal norm, social norms (injunctive, descriptive), perceived behavior control; (4) questions about trips during workhours; and (5) questions about remote meetings. For the purpose of this study, sections one, two, and three were selected and included in the analysis.

### 2.3. Measures

#### 2.3.1. Stage of Change

Building upon Bamberg’s stage-diagnostic measure aimed at car use intention [7], the stage of change construct was operationalized by a question specifically addressing statements on car use for travel to work. In Table 1, all statements and related stages are shown. In the table, the items are furthermore applied to the stages of SSBC and TTM (our interpretation). In contrast to the SSBC, we divided the predecision stage into two distinct stages that we labelled predecisional denial referring to the notion of seeing no reason to change and predecisional inhibition referring to the notion of feeling that it is impossible to change. Although the statement related to the actional stage of SSBC could be interpreted as also including preparation, we preferred to keep the original labeling as the item reflects the notion of being fully prepared for an action of change. The preactional and postactional stages were kept as suggested by the SSBC. Although the TTM, SSBC, and our stage labelling differ somewhat, it is important to note that they all follow the same stage-structure, from weaker to stronger intention to change. This is important to note since the aim of the current study was not to address each specific stage separately, but rather to test if and how the psychological mechanisms relate to being at lower or higher stages.

As can be seen in the table, 338 respondents stated that they do not travel by car. These were removed from further analyses since they cannot have an intention to reduce a car-dependent behavior. Thus, 794 respondents were retained for forthcoming statistical analyses.

#### 2.3.2. Attitude and Perceived Behavior Control

Items proposed by Ajzen [43] to capture attitudes, perceived behavioral control, and norms were used, and in line with the suggestions the questions were slightly rephrased to fit the context of car-use. Car-use change attitudes were thus operationalized as consisting of both instrumental and affective attitudes, and measured using two items phrased thus: “*Choosing a different mode to get to work would work…*” (instrumental attitude), and “*Choosing a different mode to get to work would feel…*” (affective attitude). The respondents rated these statements on 7-point Likert scales ranging from 1 (bad) to 7 (good). Perceived behavior control related to mode use change was captured using one item phrased thus: “*For me it would be easy to use another transport mode than the car to get to work*”, and answered on a 7-point Likert scale ranging from 1 (do not agree) to 7 (completely agree).

#### 2.3.3. Norms

The scale measuring personal norms concerns values, principles, and guilt with respect to not using the car to get to work, was phrased thus: “*Because of my personal values and principles, I feel an obligation/guilt in respect of not using the car to get to work*”. Social norms related to other important people in a person’s life were measured using two statements whereby the first (“*Most people that are important to me would support my choice of leaving the car and using a different mode to get to work*”) captured the injunctive social norm, while the second (“*Most people that are important to me would leave the car and choose a different mode themselves*”) captured the descriptive social norm. All the norm scales were measured on a 7-point Likert scale ranging from 1 (do not agree) to 7 (completely agree).

### 2.4. Data Analysis

An ordered logit regression model (OLM) was performed in order to understand how different factors relate to the stage of change. The OLM was chosen due to the nature of the dependent variable, i.e., the stage of change [44]. To be able to test an OLM, the independent variables must be in the form of categorical (nominal or ordinal) factors [44]. Attitudes, PBC, Personal- and Social Norms were accordingly converted into three ordinal categories: low (scale points 1 and 2 recoded to 1), medium (scale points 3 to 5 recoded to 2), and high (scale points 6 and 7 recoded to 3). The stage of change variable was coded in the sequence of predecisional denial to postactional in order to simplify interpretation of the output; as such, a higher value corresponds to stronger intention. Gender did not require any conversion (coded as women = 1, men = 2); age was recoded into three age groups of equal sizes; incomes were already categorized into three categories from low to high; household compositions were coded into four categories (cohabiting or single, with or without children); and distances were recoded into short (below 8 km), medium (8–20 km) and long distance (above 20 km). The OLM was then estimated using socio-demographic variables, instrumental and affective attitudes, perceived behavior control, personal norms, descriptive- and injunctive social norms, and with the stage of change as a dependent variable. In order to test the potential indirect effects of social norms, a multivariate linear regression was conducted later.

## 3. Results

### 3.1. Descriptives

Table 2 shows the descriptives of the 794 respondents in the analytic sample. Although representative for the worksites included in the study, there was an overrepresentation of co-habiting households and slightly higher income compared to county average.

In Table 3, the distribution of respondents in each stage of intention to change are shown. For car users, as expected, the majority categorized themselves as being in the predecisional denial and predecisional inhibition stages. This was also true for carpooling. Most respondents indicated belonging to the predecisonal inhibition stage (*n* = 275), meaning that they most frequently use the car to get to work, wanting to reduce this but feel it is impossible. The respondents in predecisional denial (*n* = 152) most frequently use the car but see no reason to change their behavior. The lowest number reported being in the actional stage (*n* = 92), with the car as their most frequently used mode but with the aim of reducing its use and knowledge of ways to take action; or in the preactional stage (*n* = 91), where they state that they most frequently use the car and plan to reduce it, but do not know when and how to change. The second largest group belong to the postactional stage (*n* = 184), indicating that they are trying to use modes other than the car as much as possible, and that they intend to maintain or reduce their already low car use in the coming months.

Of those in the early stages, we identified 2/3 as being hardcore car users who did not normally use any other mode. The remaining respondents were mixed mode users that used the car one to four days a week. For public transport, biking, and walking, the majority were, as expected, in the postactional stage. Interestingly, 46 respondents reported being in the postactional stage while using the car as their main mode. Fifteen of these were judged as misclassified since they reported using the car five days a week during the work week, in both summer and winter, and were hence removed from further analyses. The remaining 31 reported using the car for two, three, or four days a week indicating a possibility that they, although still using the car most frequently, may recently have reduced their car use, and have an intention of reducing it even more. They were judged as categorized correctly, although still being frequent car-users.

### 3.2. Ordered Logit Regression Analysis

In a first OLM, the socio-demographic variables (age, gender, income, distance to worksite, and household composition) were tested on the stages of change. The results yielded nonsignificant effects for all variables, indicating that the stages of change were independent of the socio-demographics. The socio-demographic variables were thus excluded from further analysis.

In a second OLM, personal norms, attitudes (instrumental, affective), perceived behavioral control, and social norms (injunctive, descriptive) were tested against stage of change. The results indicated that injunctive and descriptive social norms were nonsignificant. During a second iteration, the nonsignificant variables were excluded. The results showed a −2Log likelihood that the final model = 591.30, with a Chi-square of 358.59, *p* < 0.001. Nagelkerke’s pseudo-R^2^ indicates that the model explained 43% of the variance, showing a good fit with the proposed model [45].

**Figure 1:**
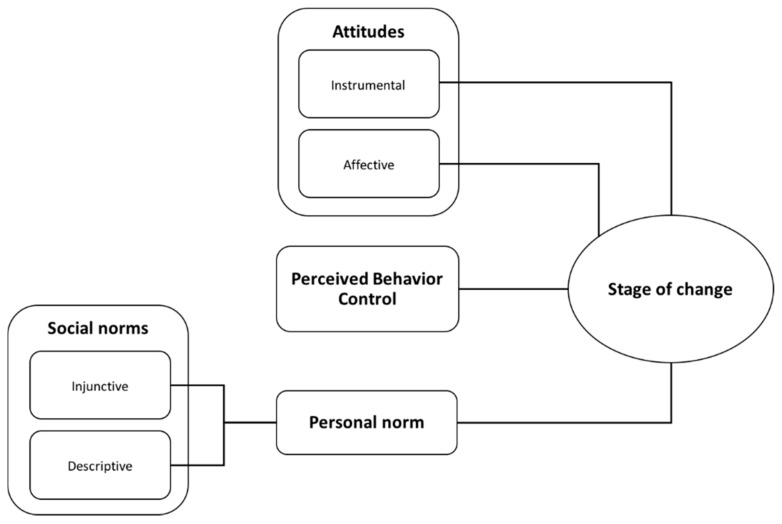
Visualization of significant relationships in Ordered Logit and Linear Regression Models.

As can be seen in Figure 1, instrumental and affective attitudes, personal norms, and perceived behavior control were all significantly related to the stage of change in the OLM. 

When assessing the ordered odds estimates of each psychological mechanism, the high ordinal category was used as reference category. The analyses are displayed in Table 4. As can be seen, affective attitude was found to be significant. The negative ordered odds estimate indicates that it is more likely to hold low or medium levels of affective attitudes toward travel behavior change at the lower stages.

For instrumental attitude, only a low level was found to be significant. No significant differences between the medium and high levels of instrumental attitude were observed, indicating a non-linear relationship between the degree of instrumental attitude and the stage of change at lower levels. The negative ordered odds estimate indicated that it is more likely to hold a low level of instrumental attitude at the lower stages.

The personal norm—that is, feeling less obligation/guilt with respect to not using the car—was also found to be related to a greater probability of being in a lower stage of change, but again only for those reporting a low level of personal norm.

Perceived behavior control showed a similar pattern, where the negative relationship with the stage of change was observed only for those reporting low perceived behavior control. Thus, the rating of easiness/being in control is related to the greater probability of being in a lower stage of change for those reporting low perceived behavior control. No difference was observed between medium and high perceived behavior control.

### 3.3. Multivariate (Linear) Regression

The nonsignificant result for social norms in the ordered logit model was a surprise bearing in mind the theoretical assumptions outlined in the introduction. However, as the personal norm has been identified as internalized social norms, we will next test the possible influence of injunctive and descriptive social norms on personal norm. As shown in Figure 1, linear regression analyses reveal a positive significant effect of the two social norm variables on personal norm (F(2, 677) = 136.3, *p* < 0.001), whereby injunctive (*β* = 0.34, t = 8.61, *p* < 0.05) and descriptive (*β* = 0.26, t = 5.95, *p* < 0.05) social norms had a significant effect (overall model fit R^2^ = 0.29) on personal norm. This result would thus speak in favor of the indirect effect of injunctive and descriptive social norms on the stage of change via personal norm [21,32].

## 4. Discussion

Applying a stage-based approach allowed us to identify car users at different stages of change. There were car users with no intention of changing, as well as car users with stronger intentions of changing. Commuters who were highly motivated could be described as multi-mode users, which, confirming previous research [40,43], implied that they were more likely to use public transport and their bikes, in addition to taking the car. Interestingly, 31 respondents reported being at the postactional stage while still using the car as their main mode. This could be an indication that they have started changing their behavior and reduced their car use, but that they are still using the car as their primary mode (i.e., they may have reduced their use from every day to three or four days a week). If one wants to identify any potential changes, it will thus be important to understand which stage of change people are currently in, and to not primarily focus on the current frequency of the target behavior.

Another finding concerns the influence of attitudes on the level (stage) of behavioral intention. In contrast to Forward [40], we differentiated between instrumental and affective attitudes. In the present study, it was shown that affective attitude was related to the intention to change travel modes; as such, those reporting low and medium affective attitude have a greater probability of having weaker intention. Commuters in the higher stages thus tend to hold a more positive affective attitude to alternatives to the car, while those in lower stages hold the most negative affective attitudes toward alternatives. Significant results were also observed for instrumental attitude. These results confirm previous travel-related studies [46,47], where it has been shown that instrumental barriers are more important during the early stages (with respect to commuting by bike). Our findings regarding stage of intention to change related to reducing car use corroborate this, as we find that those with low instrumental (and affective) attitudes have a greater probability of being at lower stages of change. However, we also find that medium and high instrumental attitudes do not differ in their stage of change, indicating that it is not a linear relationship between the degree of instrumental attitude and the stage of change. For affective attitudes, a significantly greater probability of being at a lower stage of change was observed for medium and high levels, which is more in line with a potential linear relationship. Thus, it is important to differentiate and process both affective and instrumental attitudes when designing interventions aimed at changing travel behavior, as they may be more or less important at different stages. Health researchers have identified a number of processes to be activated during interventions aimed at changing attitudes [48]. Examples include dramatic relief, positive framing, and the re-evaluation of outcomes to be targeted during the precontemplation stage, as well as different decision-making perspectives and persuasion of outcomes to be targeted during the contemplation stage. Different interventions in support of these processes to bring about attitudinal change can include positively-framed information material, group discussions and roleplay, and presenting the pros and cons of different travel modes when commuting to work e.g., [49,50,51].

Guilt and responsibility, captured in the concept of personal norm, are important when it comes to change. This study has shown that car commuters who did not feel a sense of guilt over their current behavior, and did not feel a responsibility to act, tended to be at a lower stage of change. Processes triggering changes in personal norms might therefore be important during the predecisional denial stage. Such triggers can include reliable risk assessments and an awareness of the necessity to change, and may be important to take into account when designing interventions aimed at change. For the predecisional inhibition stage, processes such as re-evaluating how one’s actions impact both the self and the environment, and identifying personal priorities may be applicable. During the preactional stage, the improvement of personal skills and self-liberation may influence people’s sense of obligation.

In line with previous research [40] we found that perceived behavioral control was of significance for the stage of change. Specifically, those reporting low perceived behavior control had a higher probability of being at a lower stage of change. However, those reporting medium levels of perceived behavior control did not differ significantly from those reporting high perceived control, indicating that the degree of perceived behavior control was not linearly related to the stage of change. The significance of this mechanism has indeed been found in previous transport studies [19,24,25,42].

Another important factor for the stage of change was social norms, although indirectly related through personal norm. Since the injunctive social norm had a somewhat stronger effect than the descriptive social norm, we conclude that individuals feel more obligated or guilty when important persons in our surroundings support sustainable travel behavior. Göckeritz et al. [30] explain this in terms of social pressure that reminds the individual of socially acceptable behavior [52,53]. 

As a consequence, it is important to integrate social norms during interventions aimed at bringing about travel behavior change. A number of processes can be activated; examples include making social norms salient (mainly during the predecisional stages), identifying and highlighting role models (during the preactional stage) and fostering helping relationships (during the actional stage). While the social norm is sought in order to facilitate change (at least during the middle stages: i.e., preactional and actional), interventions need to be adapted in terms of their intensity and form.

### Future Research and Limitations

Although we find our results clear and straightforward, there are some limitations worth noting. The design of this study does not permit conclusions regarding the importance of psychological mechanisms at a specific stage of intention to change; what we can say is that psychological mechanisms are related to higher or lower stages, which is still important. However, designing studies establishing potential stage-specific features of these mechanisms is an important future step if we want to understand how effective travel interventions regarding sustainable work commutes might look, and whether these are dependent on different stages of intention to change. This is especially important since previous critiques of stage-based models, such as TTM, have questioned the validity related to stage-classifications and the number of stages. It is therefore essential to empirically test newly developed stage-based models, such as the SSBC by Bamberg [8], in a variety of contexts. From the present study, we know that a number of psychological mechanisms are important, and that they are sometimes not linearly related to stages. For instance, our results indicate that the degree of perceived behavior control may not be linearly related to the stage of change, confirming that some mechanisms may be more stage-related than others which may have a more linear relationship. This is important since travel behavior interventions sometimes use, for instance, perceived control mechanisms (personalized information about routes and timetables, or free trials on public transport to test and learn) as a prime target for all citizens in order to increase the sense of control in efforts to change behavior. However, if this mechanism is less important for people at some stage(s), it may be a waste of resources.

A potential limitation of the present study is that the focus was on the stage of intention to reduce car use without specifying what a new target behavior could be. The results are thus not directly comparable with studies using such a target behavior. A possibility might be that different psychological mechanisms may vary in importance for intentions addressing (i) a problem behavior (e.g., reducing car use), or (ii) a new specific target behavior (e.g., starting to use public transport or a bike); this is yet another question to address in future studies.

Based on our results, future research should focus on designing interventions tailored to stages of change by using processes stressing the psychological mechanisms identified to be important, such as affective and instrumental attitudes, perceived behavior control, personal norm, and social norms. Distinguishing between stages and processes is essential when it comes to generating reliable results, especially since previous intervention studies often lack this kind of separation [4]. The processes under discussion originate from studies made in various contexts, e.g., health, physical activity, the environment, and energy conservation (e.g., [48,49,54,55,56]). Thus, it has to be established which of the processes is most effective specifically for a sustainable work commute. 

We encourage future research to scrutinize our findings by conducting replications, including multiple item scales. In this way, we will gain a deeper understanding of the role of psychological mechanisms for the intention to change travel behavior.

## 5. Conclusions

The aim of this study was to increase our understanding of the mechanisms underlying the stage of intention to change the work commute. In the context of the sustainable work commute, we have identified four psychological mechanisms driving the stage-based intention of travel mode change. Attitudes, personal norms, and perceived behavior control had a direct effect on the stage of change, while social norms had an indirect effect via personal norms. People with positive attitudes, a strong sense of obligation, and a supportive social environment were more likely to use sustainable modes for their work commute. We conclude that travel interventions should aim to integrate processes that emphasize these psychological mechanisms.

## Figures and Tables

**Table 1:** Operationalization of stages of intention to change in the current study, related to the stages in SSBC and TTM.

Current Study			Previous Stage Models	
	*n*	Current Study	Stage Model of Self-Regulated Behavioral Change (SSBC) Bamberg, 2013 [7]	Transtheoretical Model of Change (TTM) Prochaska & Velicer, 1997 [6]
**Operationalization of stages** (adapted from Bamberg, 2013)				
Question: Which of the following statements describe your car trips to and from work? Mark the statement that best matches your current situation.		**Stage**	**Stage**	**Stage** *
*I most frequently use the car to go to work. I am pleased with this and see no reason to reduce my car use.*	152	Predecisional Denial	Predecisional	Precontemplation
*I most frequently use the car to go to work. I would like to reduce my car use but feel it is impossible.*	275	Predecisional Inhibition	Predecisional	Precontemplation and Contemplation
*I most frequently use the car to go to work. I am thinking about reducing my car use but am unsure as to how or when to do this.*	91	Preactional	Preactional	Contemplation and Preparation
*I most frequently use the car to go to work. My aim is to reduce my current car use. I know which journeys to replace, and which modes to use, but have not started to do so.*	92	Actional	Actional	Preparation and Action
*I try to use other modes than the car as much as possible, I will maintain or reduce my already low car use in the coming months.*	184	Postactional	Postactional	Action and Maintenance
*As I do not travel by car, this question does not apply.*	*338*	*No need for intention to change*	*Captives*	*Maintenance*

* This is our interpretation of how stages in TTM relates to the operationalizations of stages in the present study.

**Table 2:** Descriptives of the analytic sample (*n* = 794).

Demographic Variable	%	M	SD
Women	64.0		
Age		45.5	11.5
Monthly household income (Swedish Crowns) ^1^			
Less than 34,000	8.9		
34,000–68,000	36.4		
More than 68,000	22.2		
Missing	32.5		
Household			
Single household without children	15.2		
Single household with children	6.6		
Cohabiting households without children	30.7		
Cohabiting households with children	47.5		
Driver’s license (yes)	99.1		
Car access (yes)	99.0		
Bike access	89.3		
Distance to next bus stop/train station in km			
Under 0.2	18.3		
0.21–0.5	22.8		
0.51–1.0	15.7		
1.1–3.0	14.2		
Over 3.0	18.5		
Don’t know	10.5		
Distance between home and workplace in km			
Under 2.9	11.0		
3.0–7.9	28.2		
8.0–19.9	22.5		
20–59.9	32.1		
60–99.9	5.1		
Over 100	1.1		
Mode use			
Public Transport	8.2		
Bike	11.2		
Walk	3.3		
Carpooling	11.1		
Car	65.4		
Home office/at customer	0.8		

^1^ 10,000 Swedish Crowns is approximately equal to 950 € or 1120 US$.

**Table 3:** Frequencies and percentages (in brackets) of the main travel mode use across the stages of intention to change.

Stage of Change						
	PD-D	PD-I	PreAct	Act	PostAct	Total
Public Transport	0	6 (9.4)	3 (4.7)	6 (9.4)	50 (78.1)	64
Bike	5 (5.6)	14 (15.7)	2 (2.2)	5 (5.6)	63 (70.8)	89
Walk	3 (11.5)	6 (23.1)	1 (3.8)	2 (7.7)	14 (53.8)	26
Carpool	19 (21.6)	30 (34.1)	13 (14.8)	16 (18.2)	10 (11.4)	88
Car	123 (23.7)	217 (41.7)	70 (13.5)	63 (12.1)	46 (8.8)	520
Total	152	275	91	92	184	794

Note: PD-D = Predecisional Denial, PD-I = Predecisional Inhibition, PreAct = Preactional, Act = Actional, PostAct = Postactional.

**Table 4:** Ordered Odds Estimates, Standard Error (*S.E.*), Wald statistics, and *p*-values from the Ordered Logit Model of the stage-of-change variable.

Psychological Mechanism	Ordered Odds Estimate	*S.E.*	Wald	*p*
Instrumental Attitude				
Low	−1.69	*0.29*	33.75	<0.001 *
Medium	−0.46	*0.25*	3.37	0.066
Affective Attitude				
Low	−1.31	*0.23*	31.38	<0.001 *
Medium	−0.46	*0.18*	6.23	0.013 *
Perceived Behavior Control				
Low	−0.99	*0.27*	13.33	<0.001 *
Medium	−0.33	*0.24*	1.91	0.167
Personal Norm				
Low	−1.08	*0.24*	19.52	<0.001 *
Medium	−0.30	*0.23*	1.67	0.197

Note: All variables were coded as low, medium and high, where the high category was used as reference for each variable in the analyses; * Significant at *p* < 0.05.
